# Prevalence of Migraine in General Spanish Population; Factors Related and Use of Health Resources

**DOI:** 10.3390/ijerph182111145

**Published:** 2021-10-23

**Authors:** Alejandro Salazar, Laura Berrocal, Inmaculada Failde

**Affiliations:** 1 The Observatory of Pain, University of Cádiz, 11009 Cádiz, Spain; inmaculada.failde@uca.es; 2 Research Unit, Biomedical Research and Innovation Institute of Cádiz (INiBICA), Puerta del Mar University Hospital, University of Cadiz, 11009 Cádiz, Spain; 3 Department of Statistics and Operational Research, University of Cádiz, 11510 Puerto Real, Spain; 4 Preventive Medicine and Public Health Area, University of Cádiz, 11009 Cádiz, Spain; laura_14medina@hotmail.com

**Keywords:** migraine, mental comorbidity, health services, healthcare services, headache

## Abstract

Migraine is a common neurological disorder considered the second most disabling condition worldwide. Its prevalence ranges from 2.6% to 21.7% in population studies. This study aimed to know the prevalence of diagnosed and undiagnosed migraine in the general Spanish adult population, their health care use, and factors related. A descriptive cross-sectional study was undertaken with 23,089 individuals >15 years from the 2017 Spanish National Health Survey. Three groups were defined: people diagnosed with migraine (DM), people reporting undiagnosed migraine (UM) and people without migraine. Sociodemographic, clinical and use of health resources data were collected. The scales Duke Social Support Index (DSSI) and General Health Questionnaire (GHQ-12) were used. Prevalence of DM and UM were determined with 95% confidence intervals. To determine the factors associated with DM and UM, a multinomial logistic regression model was used. The prevalence of DM was 8.6% (95%CI: 8.2–9), and UM, 0.9% (95%CI: 0.8–1). People with DM more frequently visited healthcare professionals (47.8%), required more supplementary tests (86.8), had a higher percentage of hospitalization (11.3%), and used emergency services (45.1%). Women had nearly three times the risk of DM and UM. Worse mental health was a risk factor for UM (OR = 1.20) and DM (OR = 1.18). The greater the work stress, the greater the risk of DM (OR = 1.12). An adequate monitoring and management of migraine in people with these characteristics could contribute to improving their quality of life and reducing costs in the system.

## 1. Introduction

Migraine is a common neurological disorder that, according to the data of the Global Burden of Diseases Study 2019, is considered the second most disabling condition worldwide, being responsible for 42.1 million years lost to disability (YLD) [1].

Prevalence of migraine in population studies has been reported to range between 2.6% and 21.7%, with an average of 12–14% [2]. Likewise, some differences have been found between continents with data showing a prevalence of 10.4% in Africa, 10.1% in Asia, with substantial differences among countries (from 3.1% in Singapore to 22.8% in India), 11.4% in Europe and between 12.8% and 16.4% in Central and South America [1,3,4,5,6].

In Spain, the estimated prevalence of migraine in 2016, according to the data from the Spanish National Health Survey, was 11.02%. It was higher than the prevalence in 2003 (6.54%) and similar to the prevalence in 2012 (9.69%) and 2009 (10.79%) [7,8].

The time trend prevalence of migraine in Spain is also reported by Fernandez de las Peñas et al. [9] from 2003 to 2012, who confirm an increase in this period. This result is in agreement with those found in Norway by Linde et al. [10] and has been related to the changes in social environmental, sedentary life, higher stress, unhealthy lifestyle habits or poor self-perceived health status [8,9]. However, the evolution of the disease and the factors associated with it need to be better studied.

There is evidence that migraine occurs twice as much in females compared to males (13.8% and 6.9%, respectively) and most frequently affects the working-age population [11], leading to a greater labor, personal and societal impact and causing greater consequences on family activities and on relationship with partners and children [12].

On the other hand, several authors have shown that migraine is frequently associated with many medical comorbidities including psychiatric disease. Around 40% of people with migraines report depression, which is almost twice as common in people with migraine compared to the general population [13]. Furthermore, a cumulative lifetime incidence of anxiety around 50% has been shown in these patients, and the prevalence of anxiety increases when migraine and depression come together [14,15,16].

Some studies have also revealed that patients with migraine and coexisting psychiatric disorders have poorer treatment outcomes and increased disability as compared to migraine without these comorbidities [17]. In view of this, it seems clear that there is a need to identify and properly address these problems in patients with migraine.

With respect to cost, several studies have shown that migraine produces an extensive burden related to healthcare and treatment [18] and indirect cost related to absenteeism and presentism [19]. Indicatively, in a study carried out in Spain in 2004 by Badia et al. [20], the authors found out that the economic burden of migraine was around 1076 million euros. The direct costs represented 32.0% of the total burden (344 million euros), 39.2% being for primary care visits, 28.7% for specialist visits, 20.5% for emergency room visits and a further 11.7% for migraine-specific prescription drugs. Similar results have been reported by Darbà and Marsà [21] in a recent study based on data from 2011 to 2016. These authors also found that headache disorders summed a total annual cost of 10,716,086 euros and migraine alone represented 7,302,718 euros of the total annual cost.

In view of the magnitude of the migraine and the paucity of information about the comorbidity related to this problem in Spain, we carried out the present study with the objectives: (a) to know the prevalence of diagnosed migraine (DM) and undiagnosed migraine (UM) in the general adult population in Spain; (b) to know the health care use in these subjects; (c) to analyze the sociodemographic and health factors related to DM and UM in this population.

## 2. Materials and Methods

A descriptive cross-sectional study was carried out based on data from the 2017 National Health Survey, performed by the Spanish National Institute of Statistics (INE, for its acronym in Spanish) [22]. This survey constitutes the main source of information on the health perceived by the general population in Spain. By means of a stratified three-stage sampling, a total of 23,089 individuals over 15 years old residing in the Spanish territory were interviewed.

Information was collected through a questionnaire conducted in a computer-assisted face-to-face personal interview (CAPI) between October 2016 and October 2017.

For the purposes of this study, in order to define the population that suffered from migraine in the last 12 months, 2 questions from the questionnaire were used: “Have you suffered migraine in the past 12 months?” and “Has a doctor told you that you suffer migraine?” From the information obtained in these questions, 3 groups of individuals were defined. First, the group of people diagnosed with migraine (DM) (n = 1991), who were those who answered affirmatively to both questions. Second, the people who reported undiagnosed migraine (UM) (n = 208), who were those who answered affirmatively to the first one, but not to the second one. Third, the group without migraine (NoM) (n = 20,890), who answered negatively to both questions.

In addition to sociodemographic characteristics, information on work stress (scale from 1: “no stressful” to 7: “very stressful”), satisfying job (scale from 1: “no satisfactory” to 7: “very satisfactory”), difficulties in carrying out daily activities, the state of health perceived in the last 12 months, and information on the use of health resources was collected. Particularly, visits (and number of visits) to healthcare professionals (either family/general practitioner or specialist) in the last 4 weeks, hospital admission (and number of admissions) in the last 12 months (excluding birth), waiting list for the last admission, use of emergency services in the last 12 months and supplementary tests were analyzed.

The social support perceived by the respondents was collected using the Duke Social Support Index (DSSI), validated and adapted into Spain by Bellón et al. [23] This self-administered scale is composed of 11 items scored from 1 to 5, with 1 being: “much less than I want”, 2: “less than I want”, 3: “neither much nor little”, 4: “almost as I wish” and 5: “as much as I wish”. A score lower than 32 indicates low social support, and 32 points or more indicates normal social support.

The General Health Questionnaire 12 (GHQ-12) was used to detect possible mental health disorders. This scale consists of 12 items evaluated from a dichotomous score (0-0-1-1), and an overall score ranges from 0 (best mental health) to 12 (worst mental health) is obtained by adding the items. It has shown adequate psychometric characteristics in both the general and clinical population [24].

A descriptive analysis was carried out. The prevalence of DM and UM were determined along with their 95% confidence intervals. Normality was tested with the Kolmogorov–Smirnov test. The Chi-squared, likelihood ratio, Kruskal–Wallis and Mann–Whitney tests were used to analyze the differences among groups.

To determine the factors associated with DM and UM, a multinomial logistic regression model adjusted by steps was carried out. In this analysis, the dependent variable was the presence of migraine, taking the NoM group as the reference group, and showing the results for the DM and UM groups. The criteria for independent variables to be included were both clinical and statistical, in view of the literature and the results obtained in the bivariate analyses. The results were considered statistically significant for two-tailed *p*-values lower than 0.05.

The analyses were carried out using IBM SPSS Statistics 23 and Epidat 3.1 software.

## 3. Results

### 3.1. Characteristics of the Sample and Prevalence of Migraine

The total number of respondents was 23,089, 54.1% women and 45.9% men. The mean age was 53.4 years (SD = 18.9). The prevalence of DM was 8.6% (95% CI: 8.2–9) and the prevalence of UM was 0.9% (95% CI: 0.8–1).

### 3.2. Sociodemographic and Clinical Factors Related to Migraine

We observed a higher proportion of women in both the diagnosed and undiagnosed migraine groups and a lower mean age in the UM group. Most people with DM were 45–59 years (31.6%), while people with UM were mostly 30–44 years (33.7%). A higher proportion of separated and divorced persons had UM, compared to the other two groups. The highest levels of difficulties performing daily activities were observed in the DM group (Table 1).

In the UM group, 9.6% reported a bad or very bad health status, compared to 21.9% in the DM group. The level of pain in the last 4 weeks was higher in people with DM (Table 1).

We observed that work stress was higher in the DM group (Mean = 4.7, SD = 1.7), followed by the UM group (Mean = 4.5, SD = 1.7). In addition, the group that expressed the greatest job satisfaction was the one that did not suffer from migraine. On the other hand, mental health was worse in the group with DM (Mean = 3, SD = 3.7), compared to the other two groups. However, respondents with UM reported less social support (Mean = 45.9 on the DSSI scale, SD = 7.8) compared to people with DM (Mean = 46.4, SD = 7.8) and NoM (Mean = 48, SD = 7.2). However, no significant differences were observed between UM and DM (Table 1).

### 3.3. Health Resources Factors Related to Migraine

We observed that 7679 people (33.3%) visited a health professional. Family or general practitioners had been visited in the last four weeks by 30.5% (one visit) and 6.8% (two or more visits). A total of 2058 (8.9%) had been hospitalized in the last 12 months (excluding births), and 543 (26.7%) had to be on the waiting list before admission. In 21.4% of the cases, two or more admissions for the same patient were necessary. The emergency services were used by 29.9% of the sample. 

People with DM were who more frequently visited healthcare professionals (47.8%), compared to the other groups. Family or general practitioners were more frequently visited by people with DM (49.4%), compared to people with UM (35.4%) and NoM (36%), and a similar situation was observed for specialists (31.2%, 30.1% and 22.9%, respectively). Likewise, the group with DM was the one that required more supplementary tests (86.8), higher percentage of hospitalization (11.3%), and emergency services (45.1%), compared with the other groups (Table 2).

### 3.4. Factors Related to the Presence of Diagnosed and Undiagnosed Migraine. Multinomial Logistic Regression Model

Regarding the results of the multinomial logistic regression model, in the UM group, we observed that aging was a protective factor against the presence of the disease (OR = 0.98), that is, the older the age, the lower the risk of UM. Women had nearly three times the risk of migraine than men, both diagnosed and undiagnosed. Worse mental health (according to the GHQ12 score) was also a risk factor for UM (OR = 1.20) and DM (OR = 1.18). Similarly, the greater the work stress, the greater the risk of DM (OR = 1.12), but this factor was not significant in the case of UM (Table 3).

## 4. Discussion

The study shows that the prevalence of DM in the general Spanish population is 8.6% and almost 1% of the population refers to UM. Furthermore, women have nearly three times the risk of migraine than men, both diagnosed and undiagnosed. Worse mental health and greater work stress were factors related to migraine. Finally, it should be noted that, in patients with DM, the use of health services was greater than those without migraine, in terms of medical consultations, supplementary tests, use of emergency services and hospital admissions.

Even though the prevalence observed in this study is within the range described by other authors in Spain [8], it is higher than the prevalence found in Finland [25], but lower than in Sweden [19], India [6] or the USA [4]. In Spain, Fernández de las Peñas et al. [26], and Roy et al. [8], in studies carried out in 2013 and 2019, observed slightly higher results than ours (9.6% and 9%). However, these authors do not provide the disaggregated information according to whether migraine had been diagnosed or not.

As expected, a higher frequency of the disease was observed in women (both DM and UM) and most people with DM were 45–59 years. Migraine is a complex condition in women, and several potential reasons might be argued. On the one hand, some authors have considered hormonal changes throughout life from menarche to menopause as factors related to migraine [13,27]. There is also evidence to support underlying genetic variance to explain the risk of migraine in women. In this vein, migraine might be an autosomal dominant condition in women, though recessive in men, or transmitted by a maternally inherited factor [28]. However, other studies with twins have shown that the genetic factor is not the only one that plays a role, and other factors (including environmental factors) should be taken into account [29].

It is noteworthy that the older the age, the lower the risk of UM, according to the results of the multinomial logistic regression model. Causal relationships cannot be inferred, but it could be argued that as people age, the probability of going to the hospital increases, and UM might decrease in favor of DM as a result.

The relationship between the mental status and migraine (both diagnosed and undiagnosed) found in the study is consistent with findings previously reported. Several studies have shown a bidirectional relationship, as they share a common pathogenic mechanism, genetic basis, as well as neurotransmitters, sex hormones and stress [13,30,31].

Unlike depression, the comorbidities of anxiety and migraine have received less attention. The relationship of these two pathological processes has been analyzed in a recently published systematic review [32]. The results showed that there is a strong and consistent relationship between them, with an average OR = 2.33 (2.20–2.47) among cross-sectional studies, and an average RR = 1.63 (1.37–1.93) for cohort studies carried out among migraineurs compared to non-migraineurs or healthy participants. A variety of mood and anxiety disorders have been identified not only as comorbidities but also as factors with some impact on the migraine chronification. Therefore, the early identification and treatment of psychiatric disorders in subjects with migraine should currently be considered [30,31].

An association between work stress and migraine was found in this study. These results are consistent with those reported by Goulart et al. [14], who found this relation in middle-aged current civil servants in Brazil. These authors reported some differences by sex, with high-strain jobs being independently associated with migraine in men and low job control strongly associated with migraine in women. These authors also found low social support as a factor associated with migraine in both genders [33]. In our study, the scores obtained on the DSSI social support scale were lower in subjects with migraine vs. those who did not have this disease. However, this association did not remain after adjusting for other variables such as mental status or work stress.

The results on the differences in the use of health resources found in the study are consistent with those described by Badia et al. many years ago in Spain [20] and with results from other countries [34]. Our group has also shown that the use of healthcare resources by patients with chronic pain [35] and chronic pain and disability [36] is higher than in the general population. Factors such as pain intensity, physical and mental comorbidities [37,38], and pain associated limitations and disability [39,40] have been shown to be related to greater use of healthcare services by CP patients. In addition, lower user satisfaction has been associated with sadness and headache [36]. This is not surprising, as headache is one of the most difficult, painful processes to diagnose and manage [41], and this might eventually lead to dissatisfaction with the healthcare system and more consumption of health care resources. Satisfied patients would be more willing to cooperate with healthcare professionals, following the medical guidelines and recommendations [42], resulting in a more rational use of resources.

A potential limitation of this study is that lifestyle variables, such as sedentary lifestyle, tobacco or alcohol consumption, have not been included in this study. Although they were not part of our aims, other studies have identified them as risk factors, and they should be taken into account in future studies. Another limitation might be the accuracy of migraine diagnosis. It is not the diagnosis itself but the affirmation by the respondent that they have such a diagnosis. There is no way to prove reliably that they actually have it, but the question is very clear, and they have the chance to answer separately if they suffer it and if they have a diagnosis for it, which should avoid an overestimation of DM. As a strength, apart from the large sample size and the methodology, this study adds the comparison of three groups, including diagnosed and undiagnosed migraine separately. 

## 5. Conclusions

In conclusion, the prevalence of migraine is high in the general Spanish population, and it implies a greater use of health services. Risk factors include having worse mental health, greater work stress and being a woman. This last factor is of particular interest since the differences between men and women are marked, and future studies should further explore the possible reasons. An adequate monitoring and management of the disease in people with the aforementioned characteristics could contribute to improving their quality of life and reducing costs in the system.

## Figures and Tables

**Table 1:** Sociodemographic and clinical factors related to the presence of migraine.

Variable	Category	NoM N = 20,890 n (%)	UM N = 208 n (%)	DM N = 1991 n (%)	*p* ^1^	*p* ^2^	*p* ^3^	*p* ^4^
Gender	Women	10847 (51.9)	150 (72.1)	1497 (75.2)	<0.001 ^5^	<0.001 ^5^	<0.001 ^5^	0.331 ^5^
Age (Years)	Mean (SD)	53.6 (19)	49.4 (19.2)	52.3 (17.6)	<0.001 ^6^	0.001 ^7^	0.001 ^7^	0.008 ^7^
Age (Groups)	15–29	2353 (11.3)	30 (14.4)	188 (9.4)	<0.001 ^5^	0.001 ^5^	<0.001 ^5^	0.004 ^5^
	30–44	4874 (23.3)	70 (33.7)	528 (26.5)				
	45–59	5503 (26.3)	48 (23.1)	629 (31.6)				
	60–74	4812 (23)	30 (14.4)	384 (19.3)				
	≥75	3348 (16)	30 (14.4)	262 (13.2)				
Marital status	Single	5359 (25.7)	67 (32.2)	462 (23.2)	0.001 ^5^	0.150 ^5^	0.001 ^5^	0.029 ^5^
	Married	11275 (54.1)	97 (46.6)	1093 (55)				
	Widow(er)	2694 (12.9)	30 (14.4)	248 (12.5)				
	Separated	505 (2.4)	3 (1.4)	49 (2.5)				
	Divorced	1020 (4.9)	11 (5.3)	137 (6.9)				
Laboral status	Active	9007 (43.2)	99(47.8)	813 (40.9)	<0.001 ^5^	0.085 ^5^	<0.001 ^5^	0.012 ^5^
	Unemployed	2185 (10.5)	31 (15)	271 (13.6)				
	Retired/early retired	6102 (29.2)	45 (21.7)	461 (23.2)				
	Student	1226 (5.9)	12 (5.8)	71 (3.6)				
	Unable to work	474 (2.3)	3 (1.4)	105 (5.3)				
	Housework	1872 (9)	17 (8.2)	267(13.4)				
Difficulties performing daily activities	None	13820 (66.2)	107 (51.4)	673 (33.8)	<0.001 ^5^	<0.001 ^5^	<0.001 ^5^	<0.001 ^5^
	A bit	2929 (14)	43 (20.7)	406 (20.4)				
	Moderate	2272 (10.9)	28 (13.5)	401 (20.1)				
	Quite	1269 (6.1)	25 (12)	317 (15.9)				
	A lot	592 (2.8)	5 (2.4)	194 (9.7)				
Health status in the last 12 months	Very good	4046 (19.4)	23 (11.1)	121 (6.1)	<0.001 ^5^	0.004 ^5^	<0.001 ^5^	<0.001 ^5^
	Good	10340 (49.5)	99 (47.6)	706 (35.5)				
	Moderate	4738 (22.7)	66 (31.7)	727 (36.5)				
	Bad	1393 (6.7)	16 (7.7)	315 (15.8)				
	Very bad	373 (1.8)	4 (1.9)	122 (6.1)				
Level of pain in the last 4 weeks	None	11414 (54.7)	61 (29.3)	475 (23.9)	<0.001 ^5^	<0.001 ^5^	<0.001 ^5^	0.037 ^5^
	Very mild	1803 (8.6)	16 (7.7)	107 (5.4)				
	Mild	2948 (14.1)	47 (22.6)	375 (18.8)				
	Moderate	3086 (14.8)	50 (24)	543 (27.3)				
	Severe	1358 (6.5)	29 (13.9)	403 (20.2)				
	Extreme	272 (1.3)	5 (2.4)	88 (4.4)				
Social support (DSSI)	Mean (SD)	48 (7.2)	45.9 (7.8)	46.4 (7.8)	<0.001 ^6^	<0.001 ^7^	<0.001 ^7^	0.381 ^7^
Work stress	Mean (SD)	4.3 (1.7)	4.5 (1.7)	4.7 (1.7)	<0.001 ^6^	0.278 ^7^	<0.001 ^7^	0.154 ^7^
Satisfying job	Mean (SD)	5.5 (1.4)	5.1 (1.6)	5.3 (1.6)	<0.001 ^6^	0.004 ^7^	0.001 ^7^	0.149 ^7^
Mental health (GHQ12)	Mean (SD)	1.3 (2.6)	2.6 (3.1)	3 (3.7)	<0.001 ^6^	<0.001 ^7^	<0.001 ^7^	0.857 ^7^

DM: diagnosed migraine; NoM: absence of migraine; SD: standard deviation; UM: undiagnosed migraine. ^1^ *p*-value for the difference among the 3 groups; ^2^ *p*-value for the difference between NoM and UM; ^3^ *p*-value for the difference between NoM and DM; ^4^ *p*-value for the difference between UM and DM; ^5^ Pearson’s Chi-squared; ^6^ Kruskal–Wallis H test; ^7^ Mann–Whitney U test.

**Table 2:** Health resources factors related to migraine.

Variable	Category	NoM N = 20,890 n (%)	UM N = 208 n (%)	DM N = 1991 n (%)	*p* ^1^	*p* ^2^	*p* ^3^	*p* ^4^
Visit to healthcare professionals	Yes	6649 (31.8)	78 (37.5)	952 (47.8)	<0.001 ^5^	0.081 ^5^	<0.001 ^5^	0.005 ^5^
Nº visits to a family or general practitioner in the last 4 weeks	None	10935 (63.9)	108 (64.7)	913 (50.6)	<0.001 ^5^	0.004 ^5^	<0.001 ^5^	0.001 ^5^
	1	5099 (29.8)	39 (23.4)	676 (37.5)				
	2 or more	1066 (6.2)	20 (12)	215 (11.9)				
Nº visits to a specialist in the last 4 weeks	None	9353(77.1)	95(69.9)	961 (68.7)	<0.001 ^5^	0.047 ^5^	<0.001 ^5^	0.576 ^5^
	1	2196 (18.1)	29 (21.3)	340(24.3)				
	2 or more	585 (4.8)	12 (8.8)	97 (6.9)				
Hospital admission in the last 12 months (excluding birth)	Yes	1821 (8.7)	12 (5.8)	225 (11.3)	<0.001 ^5^	0.133 ^5^	<0.001 ^5^	0.014 ^5^
Nº hospital admission in the last 12 months (excluding birth)	1	1436 (79)	9 (75)	156 (69.3)	0.002 ^5^	0.692 ^6^	<0.001 ^5^	0.672 ^6^
	2 or more	365 (20.1)	3 (25)	69 (30.7)				
Waiting list for the last admission	Yes	469 (26.1)	5 (41.7)	69 (30.9)	0.157 ^5^	0.246 ^6^	0.126 ^5^	0.446 ^6^
Use of emergency services in the last 12 months	Yes	5932 (28.4)	71 (34.1)	898 (45.1)	<0.001 ^5^	0.068 ^5^	<0.001 ^5^	0.002 ^5^
Supplementary tests	Yes	16668 (79.8)	170 (81.7)	1729 (86.8)	<0.001 ^5^	0.488 ^5^	<0.001 ^5^	0.041 ^5^

DM: diagnosed migraine; NoM: absence of migraine; UM: undiagnosed migraine. ^1^ *p*-value for the difference among the 3 groups; ^2^ *p*-value for the difference between NoM and UM; ^3^ *p*-value for the difference between NoM and DM; ^4^ *p*-value for the difference between UM and DM; ^5^ Pearson’s Chi-squared; ^6^ likelihood ratio.

**Table 3:** Factors related to the presence of diagnosed and undiagnosed migraine. Multinomial logistic regression model.

Migraine	Variable	*p*-Value	OR (95%CI)
UM	Age (Years)	0.001	0.98 (0.96–0.99)
	Gender (Women vs. Men ^1^)	<0.001	2.81 (1.80–4.37)
	Mental health (GHQ12)	<0.001	1.20 (1.12–1.28)
	Work stress	0.791	1.02 (0.9–1.15)
DM	Age (Years)	0.176	1 (0.99–1)
	Gender (Women vs. Men ^1^)	<0.001	2.96 (2.52–3.48)
	Mental health (GHQ12)	<0.001	1.18 (1.15–1.21)
	Work stress	<0.001	1.12 (1.07–1.18)

CI: confidence interval; DM: diagnosed migraine; NoM: absence of migraine; OR: odds ratio; UM: undiagnosed migraine. Reference category of the dependent variable: NoM. ^1^ Reference category.

## Data Availability

A publicly available dataset was used in this study. It can be found on the website of the Spanish Statistics Institute (INE) at: https://www.ine.es/dyngs/INEbase/en/operacion.htm?c=Estadistica_C&cid=1254736176783&menu=resultados&idp=1254735573175#!tabs-1254736195295 (accessed on 7 June 2021).
